# Treatment of postoperative intestinal dysfunction of hirschsprung's disease based on the principle of “anorectal balance”

**DOI:** 10.3389/fsurg.2022.996455

**Published:** 2022-10-28

**Authors:** Li Tian, Chensen Ma, Zhengdong Deng, Tianqi Zhu, Xiang Zhao, Ying He, Mingfa Wei, Jiexiong Feng, Donghai Yu

**Affiliations:** ^1^ Department of Pediatric Surgery, Tongji Hospital, Tongji Medical College, Huazhong University of Science and Technology, Wuhan, China; ^2^ Hubei Clinical Center of Hirschsprung's Disease and Allied Disorders, Wuhan, China

**Keywords:** anorectal balance, hirschsprung’s disease, intestinal dysfunction, botulinum toxin, internal anal sphincter

## Abstract

**Purpose:**

Radical surgery is the most effective treatment for Hirschsprung's disease. However, some children still have symptoms of intestinal dysfunction such as constipation, abdominal distension, and recurrent enterocolitis after operation. The purpose of this study was to evaluate treatment outcomes of postoperative intestinal dysfunction in children with Hirschsprung's disease by using the principle of “anorectal balance”.

**Methods:**

The clinical data of postoperative intestinal dysfunction in children with Hirschsprung's disease in the single treatment group from July 2019 to July 2021 were retrospectively analyzed. All the enrolled children underwent botulinum toxin injection (2.5 U/kg); 3 to 6 months later, the injection was performed again; the children who had received more than two botulinum toxin injections underwent the internal sphincter myectomy. Anorectal manometry was performed routinely after operation, and abdominal distension and defecation were recorded.

**Results:**

A total of thirty children with postoperative intestinal dysfunction underwent radical surgery for Hirschsprung's disease were included in this study. Symptoms of constipation, abdominal distension and enterocolitis were improved after botulinum toxin injections in most children compared to before surgery (*P* < 0.01). After re-injection of botulinum toxin in twelve children, the frequency of defecation increased, the anal resting pressure decreased, and the clinical symptoms were relieved again (*P* < 0.05). Eleven children underwent internal sphincter myectomy, and the symptoms of constipation, abdominal distension and enterocolitis were significantly improved after the operation (*P* < 0.01).

**Conclusion:**

Botulinum toxin injection and internal sphincter myectomy based on the principle of “anorectal balance” can effectively reduce the resting pressure of the anus and relieve intestinal dysfunction, and have satisfactory clinical effect.

## Introduction

Hirschsprung's disease (HSCR) is a developmental disorder characterized by the absence of ganglion cells in the distal bowel, leading to chronic functional obstruction (1). The ultimate goal of surgical treatment is to remove the non-ganglion segment of the intestine and anastomose the innervated proximal intestine with the anus, so that the patients can defecate normally after operation.

Radical surgery is the most effective treatment for HSCR (2, 3). Although the bowel function of most children can be significantly improved after operation, some children still have symptoms such as constipation, abdominal distension, and recurrent enterocolitis after operation (4, 5). Some scholars defined this postoperative anal defecation dysfunction as anal outlet obstruction. We recently conducted a systematic study of clinical data from these children and found that their symptoms might be due to weaker proximal bowel function and greater anal resistance to defecation. When the defecation force was weaker than the resistance of the anus to hinder the excretion of stool, it would lead to poor defecation and manifest as symptoms of intestinal dysfunction. In other words, the bowel function did not match the anal function and stool was retained. The essence of the physiological behavior of defecation was that the force of the intestines and the body (the pressure formed by the diaphragm and abdominal muscles) to promote defecation exceeds the force formed by the anal sphincter to hinder defecation.

Therefore, we proposed the principle of “anorectal balance”: anorectal function was a dynamic balance system, when the intestinal propulsion and anal resistance reached a dynamic balance, which was what we called “anorectal balance”, normal defecation could be achieved. For some children with HSCR who have undergone radical surgery but could not receive further bowel resection, reducing anal resistance to achieve a balance between the bowel and anus – “anorectal balance” – might be a breakthrough point in clinical diagnosis and treatment.

## Methods and materials

### Case collection

From July 2019 to July 2021, the clinical data of children with intestinal dysfunction after radical resection of HSCR in the department of pediatric surgery of Tongji Hospital Affiliated to Tongji Medical College of Huazhong University of Science and Technology were collected, including general conditions, past surgery history, number of bowel movements per day, preoperative anal resting pressure.

### Inclusion and exclusion criteria

Patients with HSCR who presented with symptoms of intestinal dysfunction after radical resection, such as constipation, abdominal distension and enterocolitis, or who did not respond to anal dilation and drug treatment, were selected for inclusion in this study. Patients with non-HSCR who presented with constipation, abdominal distension and enterocolitis after surgery were not considered to be candidates for this study.

### Treatment procedures

All patients routinely received colonic barium angiography, anorectal manometry, and rectal mucosal acetylcholinesterase staining before surgery (Supplementary Figures S1,S2).

All operations in this study were performed by a fixed surgical team. Botulinum toxin injections were performed as follows: the patient was placed on an operation table in the lithotomy position and was under anesthesia; the anal canal was repeatedly disinfected 3 times with 0.5% vital iodine; botulinum toxin was (Botulinum toxin type A for injection, trade name: Botox; manufacturer: Allergan Pharmaceuticals Ireland; import drug registration number: S2017005) injected into the internal sphincter at the 1, 5, 6, 7, and 11 o'clock directions near the dentate line, respectively, at a dose of 0.5 U/kg/point. One week after the operation, all patients received anorectal manometry again, and the number of defecation, abdominal distension, and enterocolitis were recorded. For those patients who experienced constipation, abdominal distension, and enterocolitis about 3 months after the first botulinum toxin injection, repeat botulinum toxin injection was performed.

**Figure 1:**
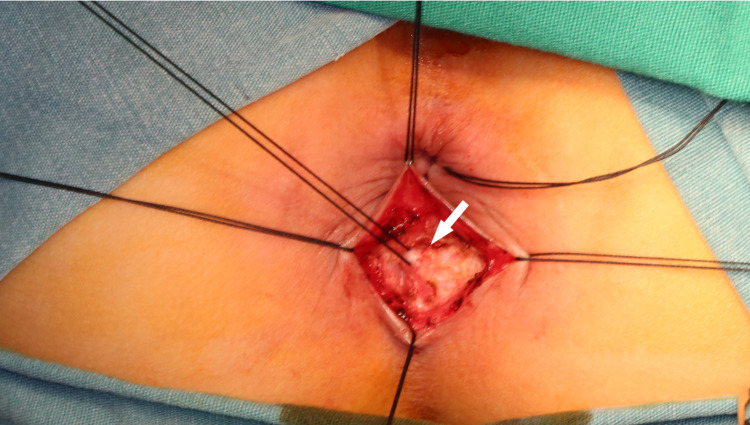
Intraoperative picture of internal sphincter myectomy. White arrow: internal anal sphincter.

According to the principle of “anorectal balance”, if receiving a second dose of botulinum toxin was effective, but the patient developed symptoms again after 3 months, the internal sphincter myectomy was performed. The patient was placed in the lithotomy position. The skin was incised in an arc at the intersphincteric groove behind the anal verge, the internal and external sphincter muscles were separated, and about a 1.5 cm-long internal sphincter strip was removed from the anal verge to the lower edge of the dentate line (Figure 1). The boundary between the internal and external anal sphincter was determined by the following criteria: (1) The external anal sphincter contracts in response to electrocautery stimulation, while the internal anal sphincter does not have this response. (2) The external anal sphincter is pink and the internal anal sphincter is white. (3) There is a layer of connective tissue between the internal and external anal sphincter muscles (6). The patients started with fluid diet on postoperative 3 day and normal diet from postoperative 5 days onwards. Anal dilatation was started from two weeks for 3 to 6 months postoperatively.

### Follow up

All patients were followed up for 3–6 months. Follow-up data were obtained by review of medical records and telephone interviews. Constipation is diagnosed by the appearance of small, pebble-like, hard stools after at least two weeks in most cases; or two or fewer stools per week (7). Abdominal distention is defined as a full abdomen with an abdominal wall higher than the line between the xiphoid process and the pubic symphysis, with or without symptoms such as vomiting and belching. Hirschsprung's disease-associated enterocolitis is defined as a patient with symptoms such as fever, diarrhea, and vomiting, and a large amount of foul-smelling stool can be excreted by digital rectal examination (1).

### Statistical methods

SPSS 22.0 software was used for statistical analysis. Measurement data were expressed as mean ± standard deviation, and enumeration data were expressed as frequency. The t test was used for measurement data, and the chi-square test and Fisher's exact test were used for enumeration data. *P *< 0.05 was statistically significant.

## Results

### Patient demographics

A total of 30 patients with HSCR were admitted to our department in the period between July 2019 and July 2021. Mean age at follow-up was 2.4 ± 1.02, and male-to-Female ratio was 1.73: 1. The main clinical manifestations of these children were abdominal distension, constipation and enterocolitis. All the enrolled children had a history of colectomy, of which 25 (83.3%) underwent subtotal colectomy and 5 (16.7%) underwent total colectomy. The primary operation had been Soave-type pull-through in 26/30 (86.7%) patients and Duhamel in 4/30 (13.3%) patients. Among them, 7 (23.3%) patients received their primary operations in our hospital, and 23 (76.7%) patients received their primary operations in other hospitals. Basic information of these children was summarized in Table 1.

**Table 1:** Demographic data and clinical characteristics (*N* = 30).

**Gender**	
Male, *n* (%)	19 (63.3)
Female, *n* (%)	11 (36.7)
Age, ¯*X* ± s, years	2.4 ± 1.02
**Clinical symptoms**	
Abdominal distension, *n* (%)	21 (70.0)
Constipation, *n* (%)	28 (93.3)
Enterocolitis, *n* (%)	23 (76.7)
**Previous operation**	
Soave, *n* (%)	26 (86.7)
Duhamel, *n* (%)	4 (13.3)
**Extent of resection**	
Subtotal colectomy, *n* (%)	25 (83.3)
Total colectomy, *n* (%)	5 (16.7)

### Outcomes after the first botulinum toxin injection

In the present study, we first compared changes in the number of bowel movements, resting anal pressure, and clinical symptoms in the enrolled children after the first injection of botulinum toxin. The results showed that after receiving botulinum toxin injection, the frequency of defecation in these children increased, the anal resting pressure was lower than that before the operation, and the clinical symptoms were significantly relieved. These differences were statistically significant (Table 2). The results suggested that botulinum toxin injection could effectively reduce anal resting pressure, thereby relieving symptoms such as abdominal distension, constipation, and enterocolitis.

**Table 2:** Outcomes after the first botulinum toxin injection (*N* = 30).

Clinical indicators	Preoperative	Postoperative	*P*	*t*/*χ*^2^
Number of bowel movements (¯*X* ± s)	1.1 ± 0.6	5.4 ± 1.1	<0.001	18.80
Anal resting pressure (¯*X* ± s, mmHg)	62.1 ± 9.1	27.3 ± 5.4	<0.001	18.01
**Clinical symptoms**				
Abdominal distension, *n* (%)	21 (70.0)	4 (13.3)	<0.001	19.82
Constipation, *n* (%)	28 (93.3)	3 (10.0)	<0.001	41.71
Enterocolitis, *n* (%)	23 (76.7)	3 (10.0)	<0.001	27.15

### Outcomes after second botox injection

It is important to note that the effects of Botox last for about 3 months. Therefore, some children will experience abdominal distension, constipation or enterocolitis again after the effect of the drug wears off. Next, we further compared the clinical parameters of 12 children who received a second botulinum toxin injection. The results showed that after receiving the second injection, the number of bowel movements increased, the anal resting pressure decreased, and the clinical symptoms were relieved again (Table 3). The results further demonstrated the effectiveness of botulinum toxin injections.

**Table 3:** Outcomes after second botulinum toxin injection (*N* = 12).

Clinical indicators	Preoperative	Postoperative	*P*	*t*/*χ*^2^
Number of bowel movements (¯*X* ± s)	1.2 ± 0.7	4.7 ± 1.2	<0.001	8.73
Anal resting pressure (¯*X* ± s, mmHg)	49.2 ± 6.3	23.4 ± 4.4	<0.001	11.63
**Clinical symptoms**				
Abdominal distension, *n* (%)	12 (100.0)	1 (8.3)	<0.001	20.31
Constipation, *n* (%)	11 (91.7)	3 (25.0)	<0.001	10.97
Enterocolitis, *n* (%)	9 (75.0)	3 (25.0)	<0.001	6.00

### Outcomes after internal anal sphincter myectomy

We performed internal anal sphincter myectomy for children who had received botulinum toxin injections twice but had recurring symptoms of abdominal distension, constipation, and enterocolitis. We found that the frequency of defecation, anal resting pressure and clinical symptoms of these children after operation were significantly improved compared with those before operation, and the difference was statistically significant (Table 4).

**Table 4:** Outcomes after internal sphincter myectomy (*N* = 11).

Clinical indicators	Preoperative	Postoperative	*P*	*t*/*χ*^2^
Number of bowel movements (¯*X* ± s)	0.9 ± 0.1	5.7 ± 1.6	<0.001	9.93
Anal resting pressure (¯*X* ± s, mmHg)	51.4 ± 3.7	17.2 ± 3.2	<0.001	23.19
**Clinical symptoms**				
Abdominal distension, *n* (%)	10 (90.9)	2 (18.2)	<0.001	11.73
Constipation, *n* (%)	11 (100.0)	2 (18.2)	<0.001	15.23
Enterocolitis, *n* (%)	9 (81.8)	1 (9.1)	<0.001	11.73

## Discussion

Surgical treatment for HSCR has been performed for over 65 years and has an overall good outcome. Nonetheless, some children complained of persistent postoperative symptoms, such as constipation, abdominal distension, and recurrent enterocolitis.

This postoperative anal defecation dysfunction has been defined as anal outlet obstruction (8). However, this definition was only a general description of symptoms and did not emphasize the function and innervation of the anal sphincter. Furthermore, the definition did not distinguish obstruction from other causes. In this study, we refered to these symptoms as postoperative bowel dysfunction. In fact, we found that some children who have undergone subtotal colectomy or total colectomy still have the above-mentioned manifestations of intestinal dysfunction, which may be due to the imbalance between the force of the bowel to promote defecation and the resistance formed by the internal anal sphincter. Therefore, we used botulinum toxin injection to relieve symptoms in some children, and performed internal anal sphincter myectomy for children who received re-injection but had exceeded the duration of the drug effect. The results show that the above treatments have achieved good clinical outcomes. Excitingly, our work is consistent with several previous studies demonstrating that botulinum toxin injections and internal sphincter myectomy can be used to treat the obstructive symptoms of patients with HSCR after pull-through surgery (4, 9).

Of note, postoperative bowel dysfunction described in this study should be differentiated from internal anal sphincter achalasia. Internal anal sphincter achalasia was also one of the causes of constipation, which was considered to be ultrashort-segment megacolon (10, 11). However, recent studies have found that internal anal sphincter achalasia was different from HSCR in clinical manifestations, pathophysiology and treatment (12, 13). The diagnostic of internal anal sphincter achalasia is determined by the following criteria: anorectal inhibitory reflex is negative, the internal anal sphincter has ganglion cells, the rectal mucosa is negative for acetylcholinesterase staining, and no surgical treatment is performed (11, 14). Although the clinical manifestations of postoperative intestinal dysfunction of children with HSCR are similar to internal anal sphincter achalasia, it is essentially a mismatch between intestinal function and anal function, that is, “anorectal imbalance”.

The essence of human defecation control is that under the control of the nervous system, the driving force of the intestine and the force of the body to promote defecation (such as the pressure formed by the diaphragm and abdominal muscles) exceed the force of the anal system to hinder defecation (such as the sphincter, pelvic floor muscles, anal pad). The internal anal sphincter provides 50% to 80% of the anal resting pressure to prevent fecal discharge (15, 16). The anorectal function is a dynamic balance system, which controls the defecation process under the coordination of the pelvic floor anal canal receptors, the primary center of the lumbosacral spinal cord and the cerebral cortex (17). Under normal circumstances, the anal resistance exceeds the defecation motivation, there is no fecal incontinence. When the feces in the intestines accumulate to a certain extent, the urge to defecate is triggered. Under the coordination of the nervous system, the force that promotes defecation exceeds the force that hinders defecation, then the feces can be discharged. In short, when the pushing force is greater than the resistance, defecation is possible; when the resistance is greater than the pushing force, defecation cannot be performed. Based on this, we propose the concept of “anorectal balance”, that is, when a dynamic balance is achieved between the intestinal propulsion and anal resistance, defecation can be achieved. In fact, the essence of treatment for children with HSCR is to remove the diseased bowel and anastomosis of the proximal normal bowel with the anus to achieve “anorectal balance”.

Some children with HSCR have symptoms such as constipation, abdominal distension, and recurrent enterocolitis because of “anorectal dysfunction”: their proximal bowel function is weaker than that of normal children, and the anal sphincter is well developed. So these children have relatively greater anal resistance. In the case of inability to further resection of the bowel with reduced function (such as subtotal colectomy or total colectomy), it is a reasonable and feasible option for clinical treatment to achieve “anorectal balance” by reducing anal resistance. In addition, we also observed that some children with HSCR had smooth bowel movements in the early postoperative period (about half a year), but gradually developed a series of symptoms such as constipation and enterocolitis 1 to 2 years after the operation. The reason may be that the initial operation will lead different degrees of damage to the anus, and the resistance of the anal sphincter will decrease in a short period of time; when the anal function is restored, the resistance formed by the sphincter will gradually increase, resulting in defecation dysfunction. Some surgeons are less disruptive to the anus during surgery based on functional protection and surgical techniques. However, it is more likely to lead to recurrence of postoperative constipation or enteritis. This further supports the applicability of the principle of “anorectal balance”.

Based on the above clues and principles, we used botulinum toxin injection and internal anal sphincter myectomy to treat postoperative intestinal dysfunction in children with HSCR, which had good clinical effect. Botulinum toxin can block the release of peripheral acetylcholine by cleaving SNAP-25 in the anterior membrane of cholinergic nerve endings, thereby leading to the relaxation of neuromuscular junctions and reducing the resistance formed by sphincter (14, 18, 19). This method has the advantages of simplicity, safety, effectiveness and repeatability. After the first injection of botulinum toxin, most of the children's symptoms were significantly relieved and no further injection was required. However, the effects of botulinum toxin only last for about 3–6 months. When the effect of the drug wore off, some children had symptoms again. Therefore, according to the principle of “anorectal balance”, botulinum toxin injection was performed again on 12 children. The results showed that while some children's symptoms disappeared within 3 months after the injection, they reappeared as the drug effects wore off. Therefore, the internal sphincter myectomy is an option reoperation when the effectiveness of botulinum toxin injection has been proven. Its essence is to achieve “anorectal balance” in anatomy and relieve the symptoms of constipation. After careful evaluation of the data of these children, we found that: (1) according to the principle of “anorectal balance”, the use of botulinum toxin injection could relieve the symptoms, suggesting that these children have high postoperative anal resistance and need to reduce the anal resting pressure to achieve the purpose of treatment; (2) the reason of some children underwent repeat botulinum toxin injections rather than direct internal anal sphincter myectomy was that the latter might lead to complications (e.g., possible fecal incontinence). Re-injection of botulinum toxin could not only further confirm the effectiveness of reducing anal resistance, but also provided a clinical basis for the implementation of internal sphincter myectomy. Recently, the author performed botulinum toxin injection and internal anal sphincter myectomy on a patient with HSCR who had recurrent enteritis and severe malnutrition after total colectomy and entero-anastomosis. About four weeks later, the child's weight increased significantly, and the symptoms were significantly relieved.

It should be noted that this study has certain limitations. On the one hand, the sample size of this study is limited, and large-scale prospective observations are still needed. On the other hand, internal anal sphincter myectomy may lead to complications such as fecal incontinence, wound infection, and prolonged healing time. Therefore, this operation needs to be performed by an experienced and skilled surgeon.

The essence of defecation control is a process in which the lower center and cerebral cortex coordinate the dynamic balance between the power of the intestine and the resistance formed by the anal sphincter. In this study, the principle of “anorectal balance” was proposed, and botulinum toxin injection and internal anal sphincter myectomy were used to treat postoperative intestinal dysfunction in children with HSCR. It can effectively reduce the anal resting pressure and relieve the clinical symptoms of the children. This may bring new ideas for the treatment of HSCR.

## Data Availability

The original contributions presented in the study are included in the article/**Supplementary Material**, further inquiries can be directed to the corresponding author/s.
