# Supplementary material for: Treatment of postoperative intestinal dysfunction of hirschsprung's disease based on the principle of “anorectal balance”
Source: Front Surg. 2022 Oct 28;9:996455. doi: 10.3389/fsurg.2022.996455 (PMC9649898; doi:10.3389/fsurg.2022.996455)
Supplement: Supplementary file 1 [file Datasheet1.pdf]

Supplementary Material

1 Supplementary Figures

1.1 Supplementary Figures

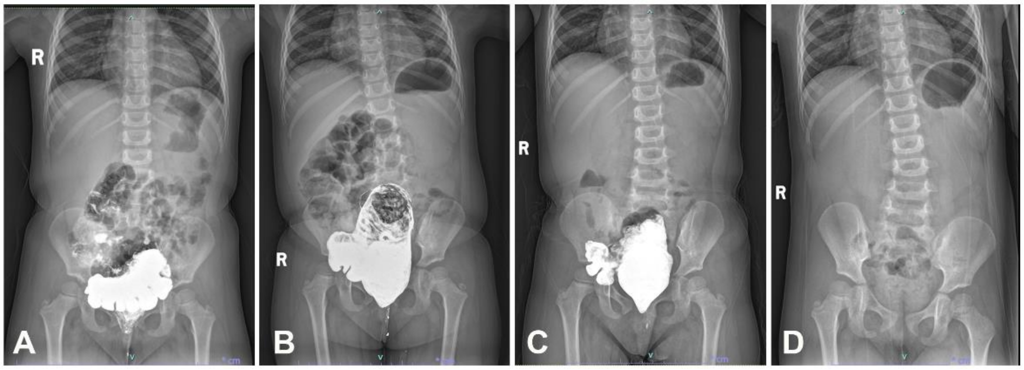

Supplementary Figure 1. Abdominal radiographs before and after internal sphincter myectomy. A, B, and C were the abdominal radiographs at 6, 12, and 24 hours after colonic barium angiography, respectively. D was the abdominal radiographs 1 week after surgery.

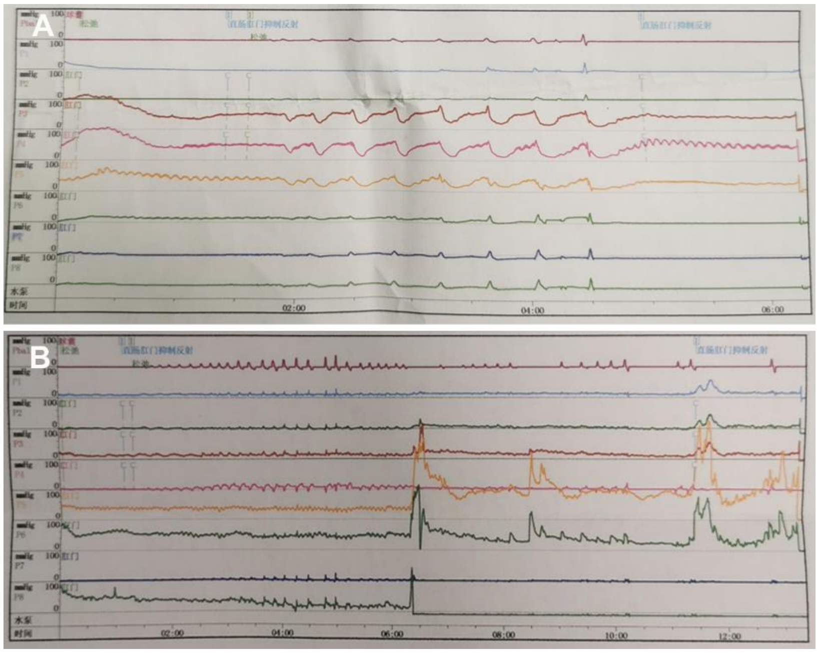

Supplementary Figure 2. Anorectal manometry before (A) and after (B) internal sphincter myectomy. Anal resting pressure: A, 44 mmHg; B, 7 mmHg.
